# Comparing DADA2 and OTU clustering approaches in studying the bacterial communities of atopic dermatitis

**DOI:** 10.1099/jmm.0.001256

**Published:** 2020-09-23

**Authors:** Christopher J. Barnes, Linett Rasmussen, Maria Asplund, Steen Wilhelm Knudsen, Maja-Lisa Clausen, Tove Agner, Anders J. Hansen

**Affiliations:** ^1^​ Natural History Museum of Denmark, Department of Biology, University of Copenhagen, Denmark; ^2^​ The Globe Institute, Faculty of Health, University of Copenhagen, Denmark; ^3^​ NIVA Denmark Water Research, Njalsgade 76, DK-2300 Copenhagen S, Denmark; ^4^​ Department of Dermatology, Bispebjerg Hospital, University of Copenhagen, Denmark

**Keywords:** atopic dermatitis, DADA2, eczema, metabarcoding, OTU clustering, skin microbiome, *Staphylococcus aureus*

## Abstract

**Introduction:**

The pathogenesis of atopic dermatitis (AD) is not yet fully understood, but the bacterial composition of AD patients’ skin has been shown to have an increased abundance of *Staphylococcus aureus *. More recently, coagulase-negative *Staphylococcus* (CoNS) species were shown to be able to inhibit *S. aureus *, but further studies are required to determine the effects of *Staphylococcus* community variation in AD.

**Aim:**

Here we investigated whether analysing metabarcoding data with the more recently developed DADA2 approach improves metabarcoding analyses compared to the previously used operational taxonomic unit (OTU) clustering, and can be used to study *Staphylococcus* community dynamics.

**Methods:**

The bacterial 16S rRNA region from tape strip samples of the stratum corneum of AD patients (non-lesional skin) and non-AD controls was metabarcoded. We processed metabarcoding data with two different bioinformatic pipelines (an OTU clustering method and DADA2), which were analysed with and without technical replication (sampling strategy).

**Results:**

We found that OTU clustering and DADA2 performed well for community-level studies, as demonstrated by the identification of significant differences in the skin bacterial communities associated with AD. However, the OTU clustering approach inflated bacterial richness, which was worsened by not having technical replication. Data processed with DADA2 likely handled sequencing errors more effectively and thereby did not inflate molecular richness.

**Conclusion:**

We believe that DADA2 represents an improvement over an OTU clustering approach, and that biological replication rather than technical replication is a more effective use of resources. However, neither OTU clustering nor DADA2 gave insights into *Staphylococcus* community dynamics, and caution should remain in not overinterpreting the taxonomic assignments at lower taxonomic ranks.

## Introduction

The pathogenesis of atopic dermatitis (AD) is complex, involving the immune system, the skin barrier function and the environment. Further, the bacterial composition of the skin from AD patients has been shown to differ significantly from that of controls [1], with higher *Staphylococcus aureus* abundance and lower overall bacterial richness [2]. More recently, coagulase-negative *Staphylococcus* (CoNS) species, such as *Staphylococcus epidermidis* and *Staphylococcus homidis*, were found to have strain-specific and highly potent killing potential for *S. aureus* [3]. However, it remains unknown whether changes in *Staphylococcus* species are simply a response to changed skin conditions [4] or are driving AD pathogenesis in humans. Large-cohort studies that include repeated skin microbiome sampling are ultimately required to disentangle this relationship, but have yet to be performed.

Sequencing techniques such as shotgun metagenomics and DNA metabarcoding have allowed for whole skin microbial communities to be characterized simultaneously. Shotgun metagenomics is a powerful technique used to determine strain-level differences between highly related *S. aureus* and CoNS species [5], but currently it remains prohibitively expensive for very large studies of the skin microbiome, as the vast majority of sequencing reads will be host derived [6]. Therefore skin-associated bacteria have predominantly been profiled using DNA metabarcoding [7], a method that targets a single variable region shared across bacteria for massively parallel sequencing, thereby avoiding host DNA. It is comparatively inexpensive and computationally less demanding than shotgun sequencing, but has been shown to lack the resolution to separate highly related species [6]. These previous metabarcoding studies were analysed using operational taxonomic unit (OTU) clustering approaches until fairly recently [8]. However, more recent methods such as DADA2 [9] and deblur [10] have emerged as complementary to, or an alternative approach to, OTU clustering, and have been shown to effectively reduce inflated bacterial richness to better represent mock bacterial communities, while also being better at aligning molecular datasets of larger organisms to morphological observations [11, 12]. While the community profiles derived from OTU clustering approaches are improved by having technical replication [13], running samples separately as triplicates uses resources that could be otherwise used for biological replication (i.e. sequencing more patients or having temporal replicates). A large number of biological replicates is particularly important when attempting to study complex relationships, such as the role of skin bacteria in AD. It remains unknown if the DADA2 approach reduces biases introduced by sequencing errors (and therefore variation within technical replicates) to such an extent that technical replication becomes unnecessary.

In this work we wanted to test whether results from metabarcoding data were improved by analysis with DADA2 instead of OTU clustering within the context of the skin bacteria associated with AD pathogenesis. Samples were collected from the stratum corneum from the volar forearm of eight AD patients (non-lesional skin) and five non-AD controls via tape stripping of the volar forearm, and the bacterial communities of each were characterized by performing DNA metabarcoding of the bacterial 16S rRNA region. Metabarcoding data were processed by two different bioinformatic pipelines, an OTU clustering method and using DADA2. We tested (a) whether DADA2 improved upon OTU clustering in differentiating between the skin bacterial communities of AD patients and healthy controls (referred to as ‘bioinformatic pipeline’). Samples were obtained in triplicate, and results were analysed with and without technical replication, and we tested (b) whether analysing data with DADA2 instead of OTU clustering reduced the need to perform technical replicates (referred to as ‘sampling strategy’). *Staphylococcus* and specifically *S. aureus* relative abundance has previously been observed to increase in abundance with AD [5], and here we performed a species-specific qPCR assay to quantify *S. aureus *. We assessed (c) whether DADA2 could improve the specificity of taxonomic assignments (in comparison to OTU clustering) by comparing the abundance and richness of reads assigned to the genus *Staphylococcus *, and to *S. aureus* specifically (from each bioinformatic pipeline with and without technical replication) using the results from the qPCR assay (referred to as ‘specificity’).

## Methods

### Study population

Eight adult AD patients ranging between 19 and 67 years old were included in the study, comprising five women and three men. Patients had no systemic treatment for AD or topical or systemic antibiotics for at least 3 months prior to sampling. Topical corticosteroid had been used by all patients within the last 30 days, but all had a break of at least 6 days prior to sampling. The inclusion criteria were age >18 years old and AD diagnosed according to UK criteria [14], whilst the exclusion criteria included breastfeeding, pregnancy and UV therapy within the 8 weeks prior to sampling (Table S1, available in the online version of this article). Five healthy controls were also sampled, including two men and three women ranging from 21 to 59 years old. The five controls had no history of manifestations of AD or any other skin disease.

### Tape stripping of the skin

Samples from both AD patients and controls were collected from non-lesional skin from the mid-volar forearm using tape strips (d-Squame, TX, USA; 15) Tapes were placed on the volar forearm and underwent standardized pressure using a d-Squame standard pressure instrument (d-Squame, TX, USA) for 10 s. Tapes were removed and placed in DNA/RNA-free 1.5 ml sterile tubes (Eppendorf, Germany) and placed on ice, before being moved for long-term storage at −80 °C.

### DNA extraction

DNA extraction from tapes was performed using a DNeasy Blood and Tissue kit (Qiagen, Germany) with minor modifications. Initially, tapes were placed in a lysing matrix E column (MP Biomedicals, UK) with 600 µl of ATL buffer, and underwent mechanical lysis with two periods of shaking at 30 Hz s^−1^ for 30 s using a TissueLyser II (Qiagen, Denmark). Supernatant was subsequently transferred to a clean 1.5 ml tube and incubated overnight at 56 °C with 20 µl of proteinase K. After lysis, extractions were performed as per the manufacturer’s instructions. Extracted DNA was diluted to 1 ng µl^−1^ for metabarcoding and qPCR analyses, while an extraction negative was performed in tandem with samples throughout, which consisted of substituting a tape with 1 µl of molecular-grade water.

### Library preparation

Metabarcoding was performed on the V3–V4 16S rRNA region for bacteria using 341F (5′-CCTAYGGGRBGCASCAG-3′) and reverse 806R (5′-GGACTACNNGGGTATCTAAT-3′) primers. Additionally, internal tags ranging between six and eight base pairs long were added to primers to increase the number of samples multiplexed per library. PCRs consisted of 1 µl of DNA extracts (1 ng µl^−1^) or 1 µl of extraction negative, 0.2 µl of AmpliTaq Gold (Applied Biosystems, Forster City, CA, USA), 2.5 µl of × 10 buffer, 2.5 µl of 25 mM MgCl2, 1 µl of each primer (at 25 mM µl^−1^), 0.2 µl of 25 mM dNTPs (Invitrogen, CA, USA), and cycling conditions consisting of: 95 °C for 5 min, then 36 cycles of 95 °C for 30 s, 56 °C for 30 s and 72 °C for 30 s, and a final extension of 72 °C for 10 min. Samples were analysed in triplicates, with each replicate separately tagged. Samples were pooled into three pools (each containing one of the triplicates for each sample) and purified using a QiaQuick PCR Purification kit (Qiagen, Denmark) following the manufacturer’s instructions. In order to convert samples into Illumina sequencing libraries, a TruSeq DNA PCR-Free Library Preparation kit (Illumina, CA, USA) was used as per the manufacturer’s instructions. In order to remove adapters, a final purification step was performed using AMPure XP magnetic beads (1:1.5 vol of beads to PCR product; Beckman Coulter, Inc., Denmark), before the completed library was sent for 250 base pair paired-end sequencing on the Illumina MiSeq platform (National High-Throughput Sequencing Centre of Denmark, Denmark).

### Bioinformatic analysis

A custom script was used to demultiplex libraries into samples, with reads assigned to samples when an exact match of both the forward and reverse tags was found (available from http://github.com/tobiasgf/lulu). During the demultiplexing process, adapters, primers and internal tags were also removed using CutAdapt (v1.9.1) [16], and unpaired reads below 100 bp were removed. Demultiplexed reads were deposited in the Sequence Read Archive, the National Center for Biotechnology Information (NCBI; accession number PRJNA534028), and are freely available for download.

Demultiplexed reads were subsequently analysed via two bioinformatic pipelines, the first being an OTU clustering approach as outlined in Bay *et al.* [17], and the second using DADA2 [within the R (v3.5.0) statistical computing environment [9]]. For the DADA2 approach, reads underwent further quality filtering as error rates were calculated and removed from the dereplicated reads. An initial sequence table was constructed before chimaeras were identified using the removeBimeraDenovo function. Finally, taxonomy was assigned using DADA2’s native naïve RDP Bayesian classifier against the Silva 128 database [18]. DADA2 produces alternaitve sequence variants (ASVs; Table S2), individual unique and quality-filtered sequences analogous to operational taxonomic units (OTUs; Table S3).

For both pipelines, samples were rarefied to an even sampling depth (5000 reads) (one triplicate from an AD patient and one from a healthy control was subsequently removed due to low reads counts for the OTU clustering approach only), and from the extraction negative (<100 reads). Data were processed with different sampling strategies, either with triplicates (technical replication), with OTUs or ASVs were considered present if detected in two out of the three replicates, or as single samples (randomly selected single samples from each triplicate). Finally, data were normalized by conversion to relative abundances for subsequent statistical analyses.

After rarefaction and merging of reads, there were 1011 OTUs in the triplicate dataset and 1974 OTUs in the single samples (4593 OTUs created before the merging of triplicates and rarefaction). Similarly, from a total of 3480 ASVs, there were 741 and 662 ASVs remaining in the triplicate and single samples after processing.

In order to provide species-level annotations, reads assigned to the genus *Staphylococcus* via both pipelines were compared to reference *Staphylococcus* species. Initially, entire 16S rRNA regions from 35 *Staphylococcus* species (with 2 strains of 6 of the most common species) were mined from the NCBI database. OTUs/ASVs and reference sequences were processed within Geneious (v2020.2), starting with a clustal W multiple alignment [19]. Reference *Staphylococcus* sequences were trimmed to match the amplicon region before phylogenetic construction using FastTree 2 [20]. Reads and reference sequences underwent phylogenetic reconstruction (separately for OTU clustering and DADA2 approaches) and the OTUs/ASVs identified as *S. aureus* were identified manually.

### Quantification of bacterial 16S rRNA copy numbers

Total bacterial 16S rRNA copy numbers were calculated using qPCR. Reaction and cycling conditions were as in metabarcoding, with the substitution of 1 µl of water with SYBR Green. A PCR product was diluted to 1 ng ml^−1^ before undergoing a dilution series (to 10^−9^), and dilutions of 10^−4^ to 10^−9^ were run together with samples and the extraction negative, in triplicate, on an Mx3005P qPCR system (Agilent, Santa Clara, CA, USA) within qPCR specific tube strips (Eppendorf, WA, USA). Standards were manually quality checked for tilting and linearity before samples were quantified against them [21]. Finally, samples were converted to copy numbers using mean amplicon lengths.

### Species-specific qPCR assay

For the *S. aureus* assay, the *femB* region was targeted with the forward primer 5′-AATTAACGAAATGGGCAGA-3′, reverse primer 5′-TGCGCAACACCCTGAACTT-3′ and probe FAM-5′-AGAAATTAACTGGATGGTACGCGCGAAGA-BHQ1-3′ (black hole quencher) [22]. Reactions consisted of: 2.5 µl of template DNA (3 ng µl^−1^), 900 nM of forward primer and 900 nM of the reverse primer, 2.5 µM of the probe and 12.5 µl of TaqMan universal PCR Master Mix (Thermo Fisher Scientific, Denmark), before being equilibrated to 25 µl total volume with molecular-grade water. Cycling conditions were: 50 °C for 2 min followed by 95 °C for 10 min, before alternating for 55 cycles between 95 °C for 15 s and 60 °C for 1 min, with end-point collection of fluorescence. Pure *S. aureus* DNA extract was used as the standard, which was diluted to 10^−9^ with molecular-grade water. Samples, standards and the extraction negative were run in qPCR specific tube strips (Eppendorf, WA, USA) within an Mx3005P qPCR system (Agilent, Santa Clara, CA, USA). *S. aureus* was quantified against a dilution series, prepared in 10-fold decrementing steps ranging from 10^4^ µl^−1^ to 10^9^ ng µl^−1^. Samples (from the eight AD patients and five controls) were also run in triplicate, and were only considered present in a sample if occurring in more than one of the triplicates, with the mean value used in downstream analyses. Using these criteria, all samples with positive occurrences amplified between 8 and 33 cycles.

### Statistical analyses

All statistical analyses were performed within the computing environment R (v 3.5.0; R Core Development Team, 2005) and visualized using ggplot2 [23]. To visualize the overall bacterial communities, Bray–Curtis similarity matrices were formed, before being plotted as non-metric multidimensional scaling analysis using the Vegan package [24]. Differences between AD patients and controls were initially tested using the envfit function within the Vegan package [24] before undergoing multivariate response generalized linear model analysis of variance (MGLM-ANOVA) using the MVABUND package [25]. Significant differences between AD patients and controls in each of overall bacterial richness were detected using Wilcoxon rank sum and signed rank tests using R’s native statistics package. Additionally, while corticosteroid use by AD patients was stopped 6 days prior to sampling, the effect of treatment time was correlated against bacterial richness using generalized linear modelling (GLM), and compositional effects were tested for using the envfit function.

The *Staphylococcus* community was partitioned from the overall metabarcoding community produced via both the DADA2 and OTU clustering approaches separately, and analyses were repeated (nMDS, envfit and MGLM-ANOVA), with the addition of a further Wilcoxon rank sum and signed rank test performed to test for significant variation between AD patients and controls in *Staphylococcus* relative abundance [26]. Shannon’s diversity was calculated within Vegan [24], while Berger–Parker indices were calculated using the Diverse package [27], which also underwent Wilcoxon rank sum and signed rank tests for significant variation between AD patients and controls.

Student’s paired *t*-tests were performed to check for significant differences in overall richness, and *Staphylococcus* richness and abundance associated with sampling strategy or bioinformatic pipeline, and unpaired Student’s *t*-tests were performed to test for significant differences between AD patients and controls (including qPCR data). *Staphylococcus* relative abundance and richness from each bioinformatic pipeline and sampling strategy were independently correlated against *S. aureus* abundance produced via qPCR using Spearman’s rank correlation coefficient [28], also using R’s native statistics package. Additionally, the reads annotated as *S. aureus* specifically (through phylogenetic analysis) from each approach were correlated against *S. aureus* abundance from qPCR using paired Student’s *t*-tests. Finally, differences in total 16S rRNA copy numbers between AD patients and healthy controls were analysed with a Wilcoxon rank sum and signed rank test, and correlated against *S. aureus* abundance (produced with qPCR) using a paired Student’s *t*-test.

## Results

### Community analyses

**Fig. 1.**
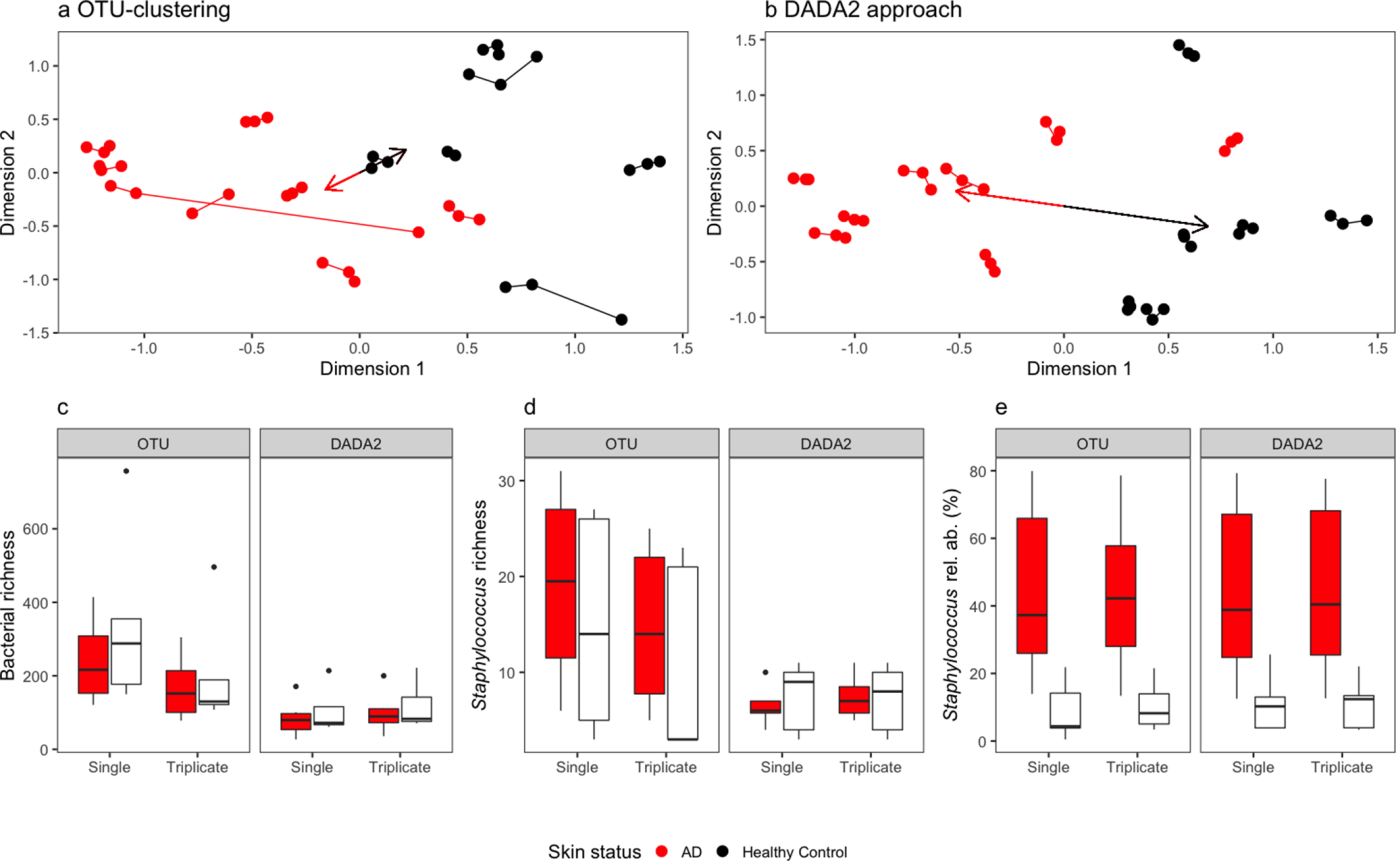
Metabarcoding of the bacterial 16S rRNA region from single tape strips was performed, singly or in triplicates (sampling strategy). Reads were processed with either (a) OTU clustering or (b) DADA2 (bioinformatic pipeline) and differences between AD sufferers and healthy controls were visualized using non-metric multidimensional scaling (with arrows pointing to group centroids). Differences in (c) bacterial richness (as OTU or ASV richness), (d) *Staphylococcus* richness (as OTU or ASV richness) and (e) *Staphylococcus* relative abundance between AD sufferers and healthy controls were visualized using boxplots.

There was consistency in the community profiles produced across bioinformatic pipelines and sampling strategies (Table 1), with the overall composition being shown to vary significantly between AD patients and healthy controls (Fig. 1a, b, Table 2), but overall bacterial richness did not (Fig. 1c). However, Shannon’s diversity measures revealed no significant differences between bioinformatic pipeline or sampling strategy. While Berger–Parker indices did not vary with replication strategy, the community profiles produced with DADA2 were significantly fewer even between both sampling strategies (Table S4). Additionally, the length of corticosteroid treatment prior to sampling did not correlate with richness or variation in community composition within AD patients in any of the datasets (Table S5).

**Table 1.** Metabarcoding of the bacterial 16S rRNA region from single tape strips was performed, singly or in triplicates (sampling strategy). Reads were processed with either OTU clustering or DADA2 (bioinformatic pipeline) and differences in OTU richness, *Staphylococcus* richness and *Staphylococcus* relative abundances between AD sufferers and healthy controls were compared using Wilcoxon rank sum tests. Differences in the overall bacterial community composition and *Staphylococcus* community compositions were assessed using envfit function within R

	DADA2				OTU			
	Single samples		Triplicate samples		Single samples			Triplicate samples
	*F*-value	*P*-value	*F*-value	*P*-value	*F*-value	*P*-value	*F*-value	*P*-value
Overall richness	15	0.524	17.0	0.724	15	0.524	18.0	0.833
* Staphylococcus* richness	17	0.712	20.5	1.000	26	0.418	28.5	0.237
* Staphylococcus* relative abundance (%)	**37**	**0.011**	**37.0**	**0.011**	**37**	**0.011**	**37.0**	**0.011**
	*R* ^2^	*P*-value	*R* ^2^	*P*-value	*R* ^2^	*p*-value	*R* ^2^	*P*-value
Overall composition	**0.356**	**0.008**	**0.355**	**0.017**	**0.440**	**0.019**	**0.457**	**0.007**
* Staphylococcus* composition	**0.617**	**0.003**	**0.616**	**0.001**	**0.135**	**0.005**	**0.570**	**0.003**

**Table 2.** Metabarcoding of the bacterial 16S rRNA region from single tape strips was performed, singly or in triplicates (sampling strategy). Reads were processed with either OTU clustering or DADA2 (bioinformatic pipeline) and differences in bacterial richness (as OTU or ASV richness), *Staphylococcus* richness (as OTU or ASV richness) and *Staphylococcus* relative abundances between single and triplicate datasets, as well as methodologies were compared using Student’s paired *t*-tests

	OTU		DADA2	
	*t*-value	*t*-value	*t*-value	*P*-value
Overall richness	−**4.775**	**<0.001**	**6.497**	**<0.001**
* Staphylococcus* richness	−1.975	0.072	1.62	0.131
* Staphylococcus* relative abundance	0.034	0.973	0.387	0.705

**Fig. 2.**
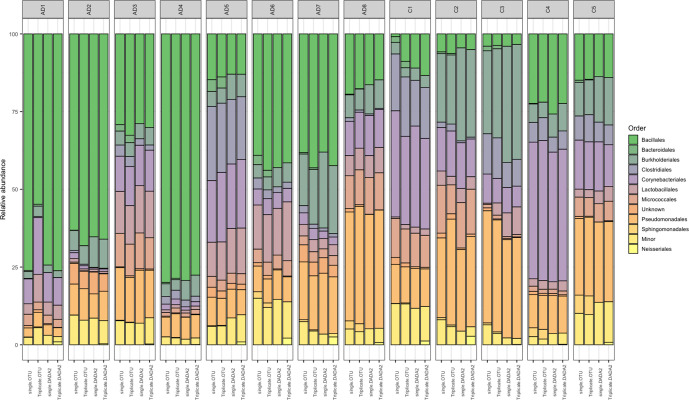
Metabarcoding of the bacterial 16S rRNA region from single tape strips was performed, singly or in triplicates (sampling strategy). Reads were processed with either OTU clustering or DADA2 (bioinformatic pipeline) and order-level relative abundances of AD patients and healthy controls were compared. For readability, orders with less than 1 % mean relative abundance were categorized as minor.

This community similarity between bioinformatic pipelines and sampling strategies was also similar in named bacterial diversity, with Bacillales, Pseudomonadales, Corynebacteriales and Burkholderiales being the most abundant orders in all approaches, averaging 31.8, 16.9, 13.7 and 10.9 % relative abundance, respectively (Fig. 2). There was, however, substantial interpersonal variation in the bacterial community at the order level, and differences between bioinformatic pipeline and sampling strategy were small in comparison (Fig. 2).

**Fig. 3.**
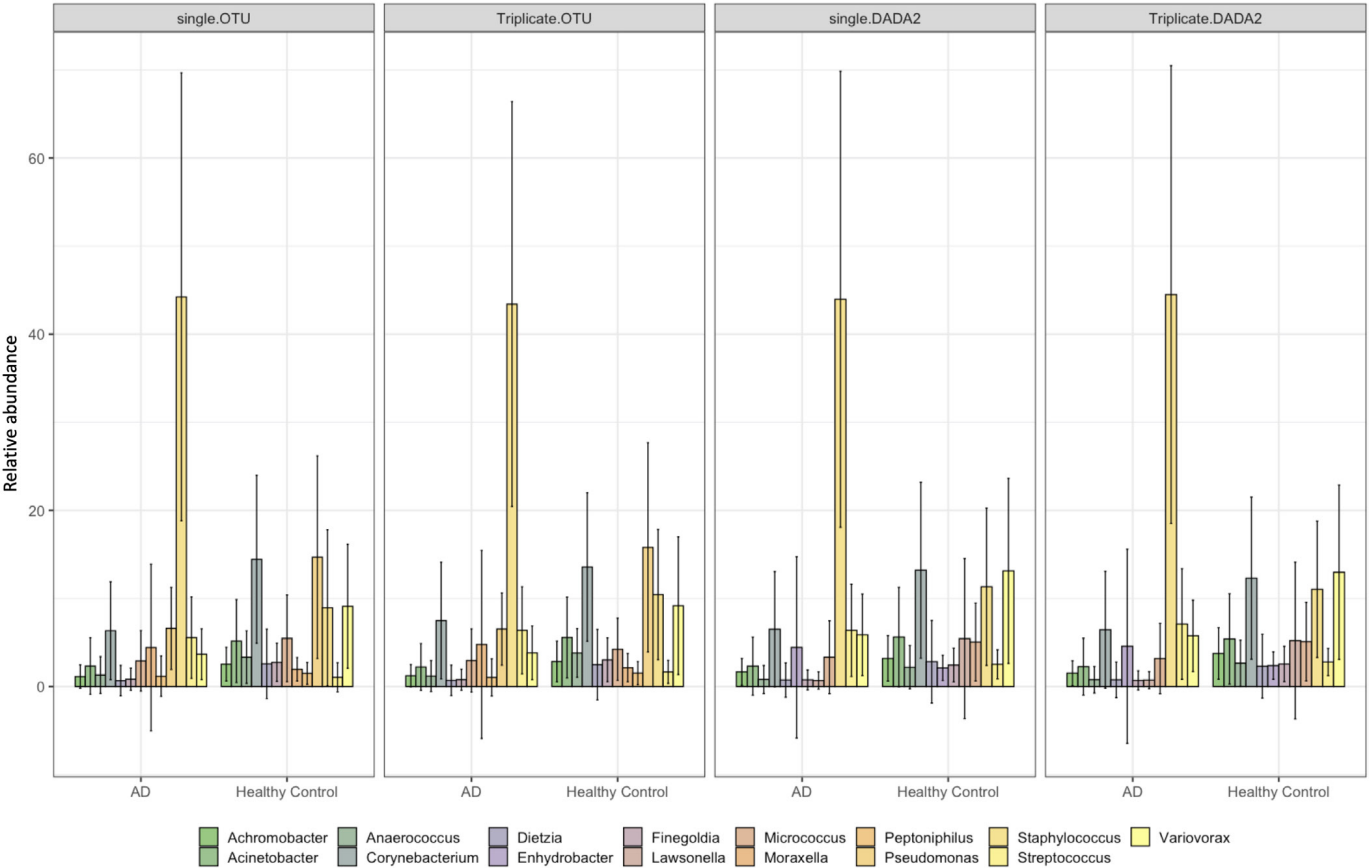
Metabarcoding of the bacterial 16S rRNA region from single tape strips was performed, singly or in triplicates (sampling strategy). Reads were processed with either OTU clustering or DADA2 (bioinformatic pipeline) and mean relative abundances of genera were compared between AD patients and healthy controls. For readability, genera with <1 % mean relative abundance were removed. Error bars represent the standard error of the mean.

There were 15 genera with a mean relative abundance of >1 % across samples, with *Staphylococcus *, *Pesudomonas*, *Cornebacterium* and *Variovorax* being the most abundant (Fig. 3), and even at this more specific taxonomic rank there were no obvious differences associated with downstream analyses, while varying considerably between individuals.

Given the importance that *Staphylococcus* species have within AD and their high abundance within our samples, the OTUs or ASVs assigned to *Staphylococcus* species were further investigated. There were 63 and 94 OTUs assigned to *Staphylococcus* species in the triplicate and single sample datasets, respectively, with only a single OTU being assigned to the species level (* S. aureus *) using the automated approach. More ASVs were assigned to the genus *Staphylococcus* in the triplicate dataset (42) than the single sample dataset (38), with 2 ASVs being assigned to *S. aureus *, 4 being assigned to *S. epidermidis* and 1 being assigned to *S. lentus* using the automated approach. Each bioinformatic technique and sampling strategy demonstrated significant differences in the *Staphylococcus* community composition between AD patients and healthy controls (Table 1). All approaches also found that that *Staphylococcus* relative abundance rose significantly with AD in comparison to the healthy controls (Fig. 1e, Table 2), although *Staphylococcus* richness did not increase significantly (Fig. 1d).

### Comparing sampling strategies and bioinformatic pipelines

The effect of sampling strategy (with and without technical replication) was compared using each bioinformatic pipeline (Table 2), revealing that richness measures significantly varied, but community compositions did not. For the OTU analysis, overall bacterial richness significantly reduced from pooling technical replicates, as mean richness fell from 239 to 165 OTUs for the AD patients, and 345 to 209 OTUs for healthy controls (Fig. 1c). However, neither *Staphylococcus* OTU richness nor *Staphylococcus* relative abundance varied significantly between sampling strategies (Fig. 1d, e). Similarly, total bacterial richness varied (ASVs) with sampling strategy when processed with the DADA2 pipeline. Conversely, technical replication increased bacterial ASV richness, from 82 to 97 ASVs in the AD patients, and 106 to 119 ASVs in the healthy controls (Fig. 1d, e). Sampling strategy did not significantly affect *Staphylococcus* richness or *Staphylococcus* relative abundances processed with DADA2.

Differences in the overall bacterial communities, *Staphylococcus* richness and relative abundance were investigated between bioinformatic pipelines (Table 3), which were remarkably consistent. The OTU clustering approach was associated with considerably increased overall bacterial richness compared to DADA2 with both sampling strategies, with means of 292 OTUs and 94 ASVs without technical replicates, and 187 OTUs to 107 ASVs with technical replication (Fig. 1c). Similarly, *Staphylococcus* richness was significantly higher when using the OTU approach compared to the DADA2 approach across sampling strategies (Fig. 1d), with means of 17 OTUs and 7 ASVs without technical replicates, and 13 OTUs to 7 ASVs with technical replication. Meanwhile, *Staphylococcus* relative abundance did not differ between sampling strategies (Fig. 1e), with higher *Staphylococcus* relative abundance found within AD treatments across all approaches (with mean relative abundances ranging from 8.9–11.3 % for healthy controls, and 43.4–44.5 % for AD patients).

**Table 3.** Metabarcoding of the bacterial 16S rRNA region from single tape strips was performed, singly or in triplicates (sampling strategy). Reads were processed with either OTU clustering or DADA2 (bioinformatic pipeline) and differences in OTU richness, *Staphylococcus* richness and *Staphylococcus* relative abundances between single and triplicate datasets, as well as methodologies were compared using Student’s paired *t*-tests

	Single sample comparisons		Triplicate comparisons	
	*t*-value	*P*-value	*t*-value	*P*-value
Overall richness	15.0	0.524	17.0	0.724
* Staphylococcus* richness	**4.485**	**0.001**	**2.759**	**0.017**
* Staphylococcus* relative abundance	−0.810	0.434	−0.504	0.623

### Comparing results to qPCR

**Fig. 4.**
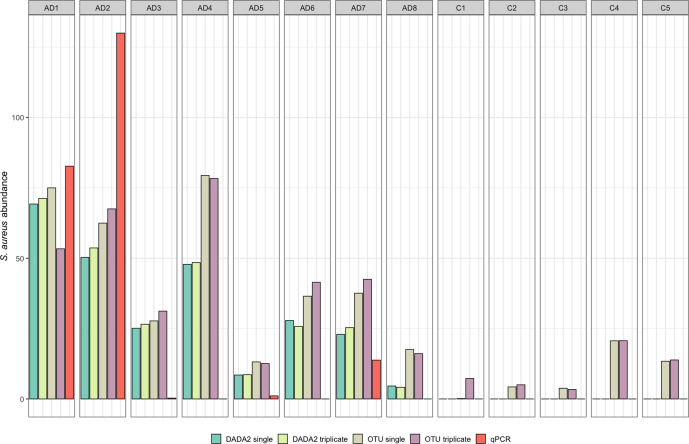
Metabarcoding of the bacterial 16S rRNA region from single tape strips was performed, singly or in triplicates (sampling strategy). Reads were processed with either OTU clustering or DADA2 (bioinformatic pipeline). *S. aureus* abundance was quantified with species-specific qPCR assay and compared to *S. aureus* relative abundance from metabarcoding datasets.

Results from qPCR revealed significantly greater *S. aureus* richness in AD patients compared to healthy controls (with no *S. aureus* found within controls; *W*=32.5, *P*=0.045), which was poorly represented by all the metabarcoding approaches. The qPCR quantification of *S. aureus* was correlated against *Staphylococcus* richness and relative abundance produced via each sampling strategy and both bioinformatic pipeline, revealing no significant correlations (Table S6). We further attempted to add high-quality species-level annotations to both datasets through phylogenetic analyses against reference *Staphylococcus* species (Figs S2 and S3), to ultimately identify *S. aureus* OTUs/ASVs. However, there were no significant correlations between *S. aureus* abundance produced with qPCR or any of the metabarcoding approaches (Table S7), with *S. aureus* falsely identified in multiple samples across pipelines, with and without replication (Fig. 4).

There was also no significant difference in 16S rRNA copy numbers between AD patients and HC controls (*W*=24, *P*=0.622), and nor was there any correlation between *S. aureus* abundance (produced with qPCR) and 16S rRNA copy numbers (*t*=−1.274, *P*=0.223) (Table S8).

## Discussion

Here we demonstrated the power of metabarcoding with universal 16S rRNA primers by replicating larger studies of AD with a limited sampling size [2], and our findings confirm significant differences in the overall bacterial and *Staphyloccocus* community compositions between AD non-lesional skin and control skin. Encouragingly, we found little difference in community-level comparisons performed with DADA2 and OTU clustering, with or without the inclusion of technical replicates. Therefore the wealth of metabarcoding studies that have investigated the role of bacteria within AD, which have mostly been performed with OTU clustering approaches, continue to provide considerable community-level insight.

However, technical replication significantly reduced the overall number of bacterial OTUs (mean of 36 % fewer OTUs per sample), and OTUs assigned to *Staphylococcus* from the OTU clustering approach (mean of 25 % fewer OTUs per sample). This suggests that sequencing errors may artificially inflate molecular richness, as has previously been noted in other environments [29]. Data analysis with DADA2 increased the number of ASVs with technical replicates (mean increase of 14 % more ASV per sample), suggesting that DADA2 accounts for sequencing errors more accurately, and is less likely to inflate bacterial richness. Rarefaction curves suggested under-sequencing in both approaches, and the increased number of reads per sample included in the triplicate analysis are most likely the reason for the increased ASV richness observed with DADA2 (Fig. S1). Given the greater consistency between technical replicates, a DADA2-based approach would most likely benefit from an increased number of biological replicates rather than technical replication, so long as adequate sequencing depth is achieved. Previously, between 10 000 and 15 000 reads per sample was suggested to generally represent over 95 % of total bacterial diversity, and the results here are in agreement with this figure [30].

The taxonomic compositions of communities were similar to previous descriptions regardless of sequencing strategy, despite potentially confounding effects of corticosteroid treatment and low sampling numbers. Communities were dominated by *Cornebacterium* and *Staphylococcus* species [31], and interpersonal variation far exceeded any impacts of variation associated with methodology. Closer inspection of the *Staphylococcus* communities revealed that AD patients and healthy controls differed significantly in composition across different metabarcoding approaches, echoing previous findings with more precise methodologies (such as culturing of bacteria [32] and shotgun metagenomics [5]), suggesting credible OTU/ASV assignments at the genus level [8]. However, crucially in this work, we highlight that metabarcoding provided inadequate taxonomic identification to investigate within*-Staphylococcus* community dynamics, as so few OTUs and ASVs were assigned to a species using automated approaches. Even with manual curation of species-level *Staphylococcus* OTU/ASV assignments, there was still a mismatch between species-specific qPCR results and *S. aureus* abundance calculated from all metabarcoding analyses. Incomplete lineage sorting and high sequence homology have previously been noted for the genus *Staphylococcus* [33], and it is therefore unlikely that any universal 16S rRNA region resolves *Staphylococcus* species effectively, no matter the bioinformatic pipeline or level of replication. However, it should be noted that the level of variation within the 16S rRNA region is highly variable between taxa, and higher taxonomic resolution may be obtained when studying other taxonomic groups with these primers [34].

The differing colonization rates between healthy controls and AD patients found within the qPCR assay mirrored results from a previous meta-analysis [35]. We therefore believe that qPCR currently represents a useful complement to DNA metabarcoding of the 16S rRNA region when studying the microbiome of AD patients. It allows for highly reliable quantification and separation of *Staphylococcus* species and probes have already been designed for CoNS species, including *S. epidermidis* and *S. homidis* [36]. The combination of qPCR and metabarcoding would allow for the screening of the very large numbers of samples over time that are probably required to disentangle the effects of *S. aureus* and CoNS species within AD in a relatively rapid and inexpensive manner, until the continuing reduction in sequencing costs and improvements to computing power make metagenomic approaches more cost-effective.

## Supplementary Data

Supplementary material 1

Supplementary material 2
