# Supplementary material for: Comparing DADA2 and OTU clustering approaches in studying the bacterial communities of atopic dermatitis
Source: J Med Microbiol. 2020 Sep 23;69(11):1293–302. doi: 10.1099/jmm.0.001256 (PMC7717693; doi:10.1099/jmm.0.001256)
Supplement: Supplementary material 1 [file jmm-69-1293-s001.pdf]

SUPPLEMENTARY MATERIAL

Title: Comparing DADA2 and OTU-clustering approaches in studying the bacterial communities of atopic dermatitis

Authors: Christopher J. Barnes PhD[1,2], Linett Rasmussen MSc[2], Maria Asplund PhD[2], Steen Wilhelm Knudsen PhD[1], Maja-Lisa Clausen PhD, MD[3], Tove Agner, PhD, MD[3] and Anders J. Hansen PhD[2]

Contents:

Figure S1 Metabarcoding of the bacterial 16S rRNA region from single tape strips was performed. Reads were processed with either OTU-clustering (blue) or b) DADA2 (orange) and rarefaction curves (read number against OTU/ASV richness) plotted for each individual triplicate plotted.

Figure S2 Phylogenetic tree comparing OTUs assigned to the *Staphylococcus* genus were compared to reference *Staphylococcus* species downloaded from the NCBI database.

Figure S3 Phylogenetic tree comparing ASVs assigned to the *Staphylococcus* genus were compared to reference *Staphylococcus* species downloaded from the NCBI database.

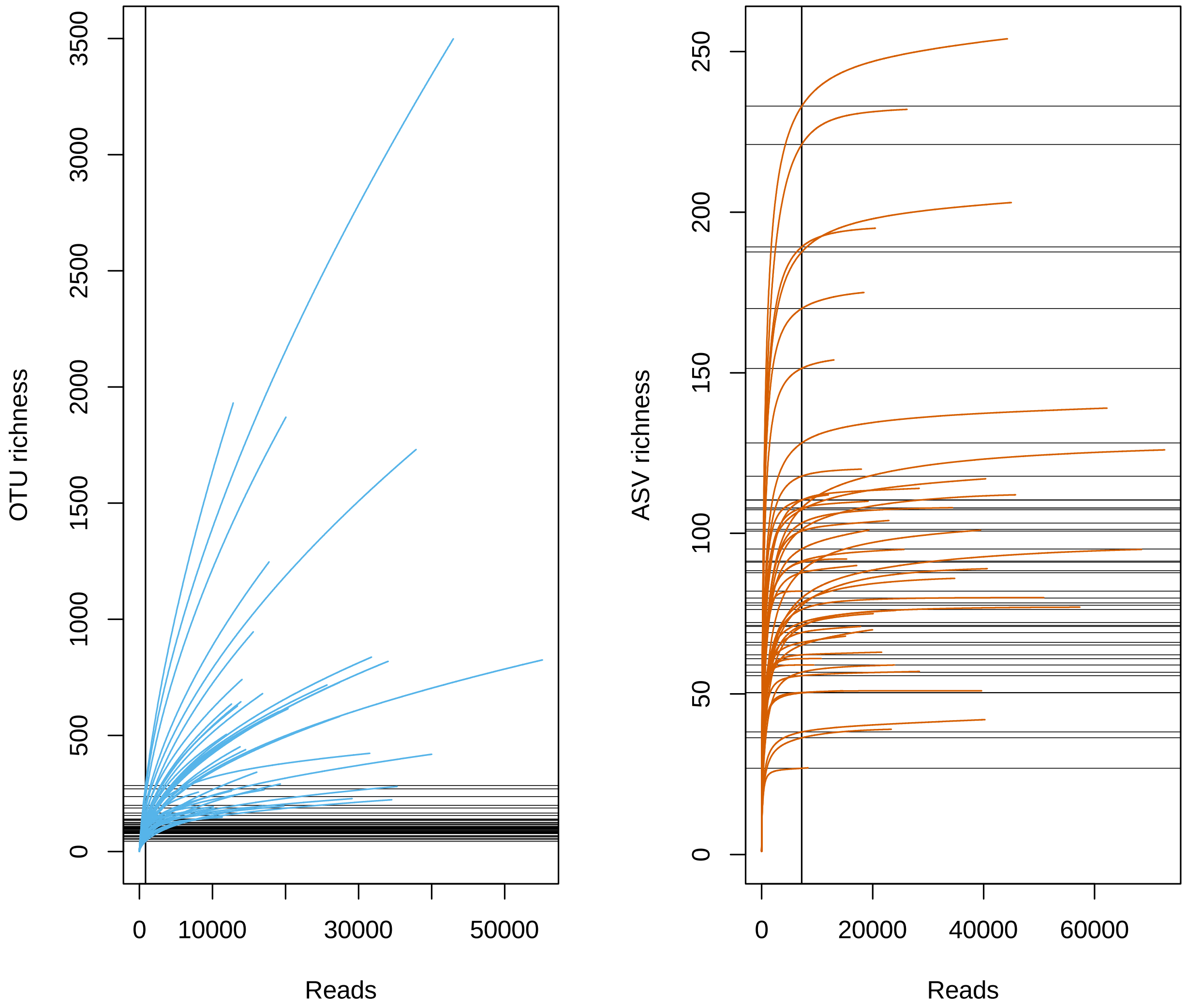

Figure S1 Metabarcoding of the bacterial 16S rRNA region from single tape strips was performed. Reads were processed with either OTU-clustering (blue) or b) DADA2 (orange) and rarefaction curves (read number against OTU/ASV richness) plotted for each individual triplicate plotted.

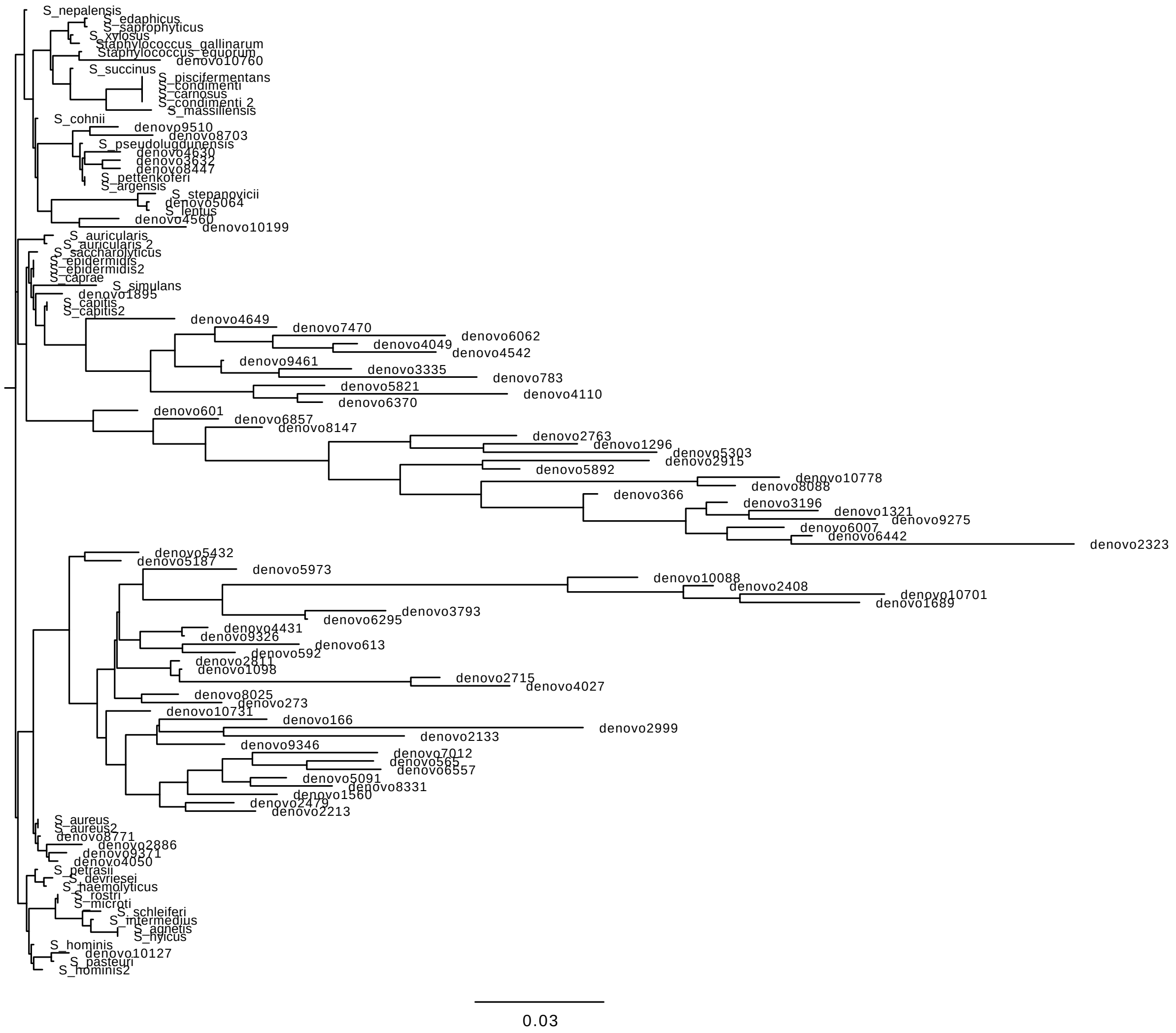

Figure S2 Phylogenetic tree comparing OTUs assigned to the *Staphylococcus* genus were compared to reference *Staphylococcus* species downloaded from the NCBI database.

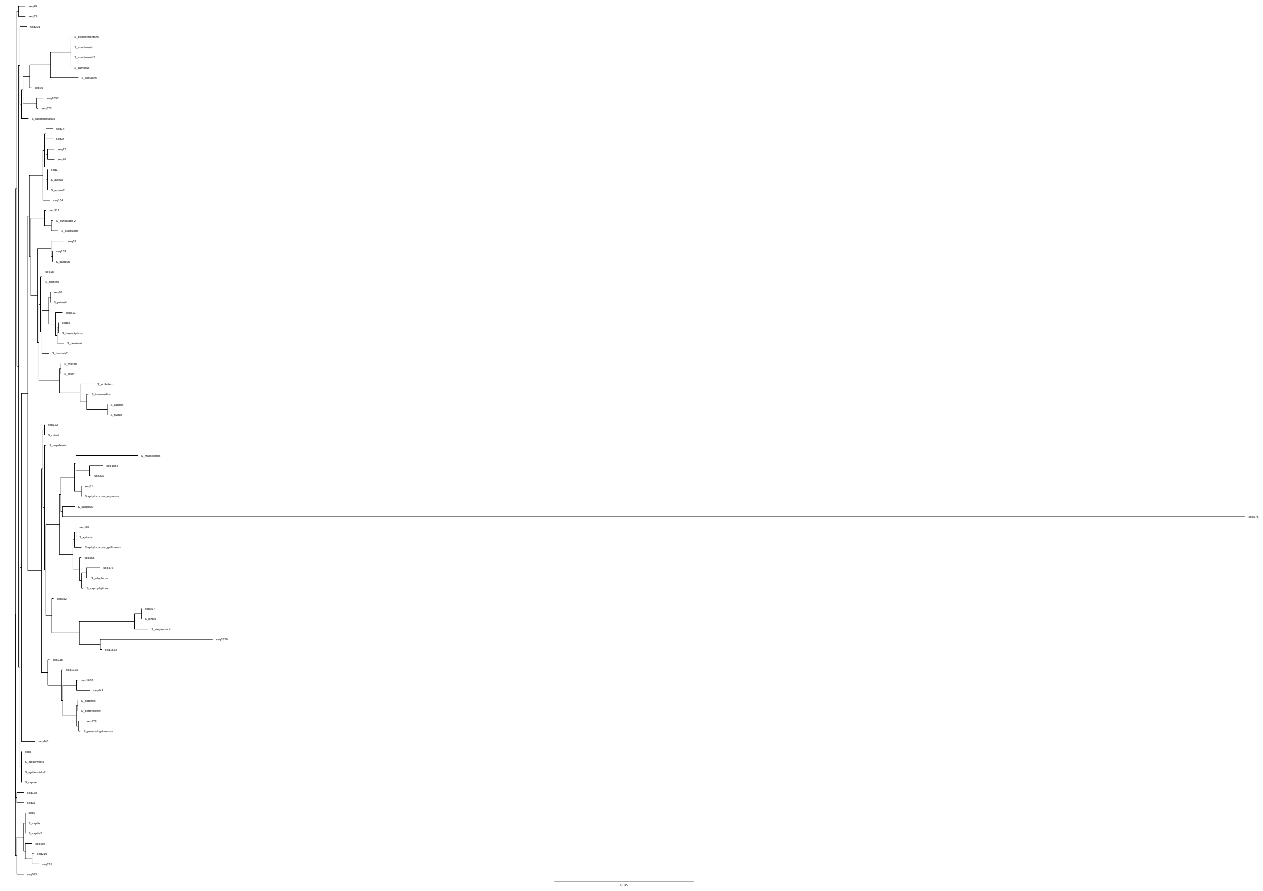

Figure S3 Phylogenetic tree comparing ASVs assigned to the *Staphylococcus* genus were compared to reference *Staphylococcus* species downloaded from the NCBI database.
